# Integrating CT-based radiomics and clinical features to better predict the prognosis of acute pancreatitis

**DOI:** 10.1186/s13244-024-01887-2

**Published:** 2025-01-09

**Authors:** Hang Chen, Yao Wen, Xinya Li, Xia Li, Liping Su, Xinglan Wang, Fang Wang, Dan Liu

**Affiliations:** 1 Department of Radiology, Yongchuan Hospital of Chongqing Medical University, Chongqing, China; 2 Department of Radiology, Chongqing Beibei District Hospital of Traditional Chinese Medicine, Chongqing, China; 3 Shanghai United Imaging Intelligence, Shanghai, China; 4 Present Address: Department of Medicine, Beth Israel Deaconess Medical Center and Harvard Medical School, Boston, MA USA

**Keywords:** Computed tomography, Acute pancreatitis, Radiomics, Prognosis

## Abstract

**Objectives:**

To develop and validate the performance of CT-based radiomics models for predicting the prognosis of acute pancreatitis.

**Methods:**

All 344 patients (51 ± 15 years, 171 men) in a first episode of acute pancreatitis (AP) were retrospectively enrolled and randomly divided into training (*n* = 206), validation (*n* = 69), and test (*n* = 69) sets with the ratio of 6:2:2. The patients were dichotomized into good and poor prognosis subgroups based on follow-up CT and clinical data. The radiomics features were extracted from contrast-enhanced CT. Logistic regression analysis was applied to analyze clinical-radiological features for developing clinical and radiomics-derived models. The predictive performance of each model was evaluated using the area under the receiver operating characteristic curve (AUC), calibration curve, and decision curve analysis (DCA).

**Results:**

Eight pancreatic and six peripancreatic radiomics features were identified after reduction and selection. In the training set, the AUCs of clinical, pancreatic, peripancreatic, radiomics, and combined models were 0.859, 0.800, 0.823, 0.852, and 0.899, respectively. In the validation set, the AUCs were 0.848, 0.720, 0.746, 0.773, and 0.877, respectively. The combined model exhibited the highest AUC among radiomics-based models (pancreatic, peripancreatic, and radiomics models) in both the training (0.899) and validation (0.877) sets (all *p* < 0.05). Further, the AUC of the combined model was 0.735 in the test set. The calibration curve and DCA indicated the combined model had favorable predictive performance.

**Conclusions:**

CT-based radiomics incorporating clinical features was superior to other models in predicting AP prognosis, which may offer additional information for AP patients at higher risk of developing poor prognosis.

**Critical relevance statement:**

Integrating CT radiomics-based analysis of pancreatic and peripancreatic features with clinical risk factors enhances the assessment of AP prognosis, allowing for optimal clinical decision-making in individuals at risk of severe AP.

**Key Points:**

- Radiomics analysis provides help to accurately assess acute pancreatitis (AP).
- CT radiomics-based models are superior to the clinical model in the prediction of AP prognosis.
- A CT radiomics-based nomogram integrated with clinical features allows a more comprehensive assessment of AP prognosis.

**Graphical Abstract:**

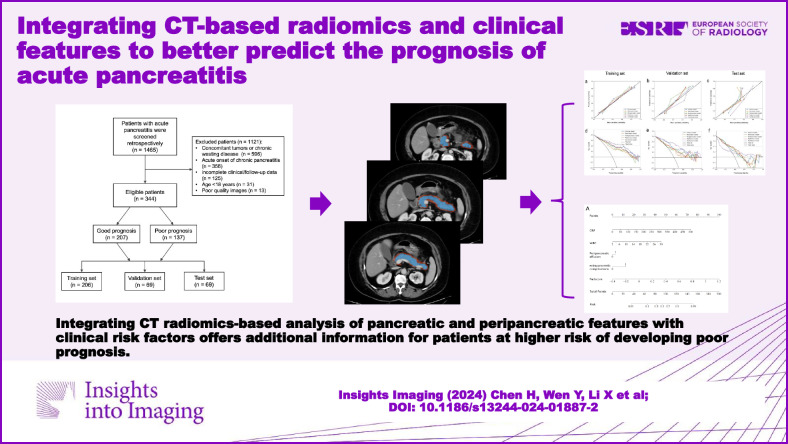

## Introduction

Acute pancreatitis (AP) is a disease characterized by a local and systemic acute inflammatory response and histological acinic cell destruction of the pancreas, which is a very frequent acute gastrointestinal disease [1]. Most cases of AP are mild with a self-limiting course; approximately 20% of patients will develop moderate or severe pancreatitis, even accompanied by necrosis of the pancreatic or peripancreatic tissue or organ failure, resulting in mortality rates ranging from 13% to 40% [2–4]. The mortality rate of AP is closely associated with its severity. Mild cases of AP generally have a good prognosis and are discharged within one week, whereas severe individuals often have multiple complications and organ failure, which is associated with high mortality [1]. Therefore, early evaluation of the prognosis in patients with AP and the improvement of the prognosis in those individuals at risk of developing severe AP is crucial.

Laboratory markers are used to indirectly infer the severity and prognosis of AP but are susceptible to interference from concomitant diseases [5]. Computed tomography (CT) has been the most common modality for directly visualizing morphological changes in the pancreas and assessing the condition of patients with AP [6], which is not performed as routine diagnostic workup of AP, but in unclear cases and/or to detect complications. However, it might not always identify necrosis in its initial stage or detect small necrotic lesions [7]. However, magnetic resonance imaging (MRI) can sensitively and non-invasively evaluate the pancreatic duct and biliary tree using magnetic resonance cholangiopancreatography (MRCP) [8], as well as detect changes in various tissue compositions [9]. However, the evaluation of CT or MRI relies on the subjective expertise of radiologists. Furthermore, a quantitative metric, the apparent diffusion coefficient (ADC) based on the pancreas, was applied to predict the progression of AP [10], but it may ignore information regarding peripancreatic inflammation. Recently, radiomics has shown the ability to identify AP patients at a higher risk of recurrence or to predict the occurrence of persistent organ failure [11, 12]. However, these radiomics studies focused on pancreatitis exhibit relatively insufficient quality and share common scientific limitations and more research on the prognosis of AP is needed [13].

Therefore, the primary aim of the current study was to develop and validate CT radiomics-based models to predict AP prognosis, and the secondary aim was further integrate radiomics with clinical risk factors to perform a more comprehensive assessment compared to conventional scoring systems.

## Methods

### Patient population

**Fig. 1:**
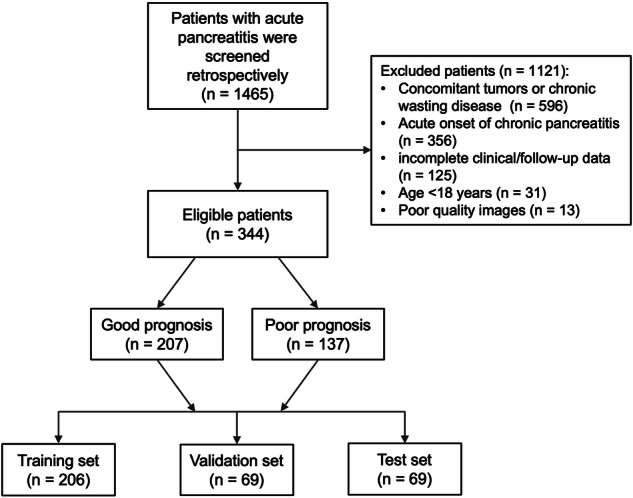
Flowchart of patient enrollment in the current study

This study received approval from the local institutional ethics committee, and the requirement for written informed consent was waived for the retrospective nature. AP patients were retrospectively screened from August 2015 to March 2022. The flowchart of patient recruitment is shown in Fig. 1. The inclusion criteria for the study were as follows: (1) patients were diagnosed with AP according to the revised Atlanta classification and definitions (2012 version) [14], who underwent upper abdominal contrast-enhanced CT scan within 72 h of symptom onset; (2) all patients were treated with fasting, acid and enzyme suppression, fluid infusion and laboratory test obtained during hospitalization; and (3) patients underwent subsequent abdominal follow-up contrast-enhanced CT scan at 7–10 days after initial CT scan according to the revised Atlanta classification and definitions (2012 version) [14]. The exclusion criteria consisted of: (1) patients were diagnosed with concomitant tumors or chronic wasting disease; (2) patients with a history of chronic pancreatitis were excluded due to the potential assessment bias induced by chronic interstitial changes (excluding acute onset of chronic pancreatitis); (3) patients with incomplete clinical data; (4) CT images of poor quality; and (5) patients with age < 18 years. The various clinical characteristics of patients, gender, age, etiology, history of diabetes, severity of AP, Bedside Index for Severity in Acute Pancreatitis (BISAP) score, white blood cell (WBC), C-reactive protein (CRP), procalcitonin (PCT) were collected. Further, the BISAP score calculation and laboratory test were conducted on all patients within 24 h of admission. The severity of AP was categorized into three degrees: mild (MAP), moderately severe (MSAP), and severe AP (SAP), in accordance with the revised Atlanta classification [14].

### Definition of prognosis

Patients were categorized into good and poor prognosis subgroups mainly based on the follow-up CT scan indicating the occurrence of pancreatic and/or peripancreatic infected necrosis or persistent (≥ 48 h) organ failure according to the previous literature and our local experience [14–17]. Categorization was performed by two radiologists (H.C. and Y.W., with 4 and 3 years of experience, respectively). The presence of infection might be indicated when extraluminal gas is observed in pancreatic and/or peripancreatic tissues on contrast-enhanced CT [14]. Organ failure was defined using the modified Marshall scoring system, and a score of 2 or more in any system indicates the presence of organ failure [16, 18]. The poor prognosis group met one of the following conditions: (1) follow-up CT showed an increase in lesion size or modified CT severity index (MCTSI); (2) there is extraluminal gas in the pancreatic and/or peripancreatic tissues on follow-up CT indicating the presence of infected necrosis [14]; or (3) the occurrence of infection or persistent organ failure during hospitalization.

### CT image acquisition

CT examinations were performed by using the commercial multidetector scanner (256-section Philips Brilliance iCT, Philips Medical Systems) covering from the diaphragm level to the inferior pole of the kidneys. The acquisition parameters for the CT images were as follows: tube voltage, 120 KV; tube current, auto mAs; layer thickness, 5 mm; layer spacing, 5 mm; pitch, 0.984–1.375; field of view, 300 × 400 mm; matrix size, 512 × 512. In addition, iohexol (350 mgI/mL) was administrated intravenously through a peripheral vein at a flow rate of 3.0–4.0 mL/s with a dose of 1.5 mL/kg [19], using a pressure syringe. The arterial phase images were obtained with a post-injection delay of 25–28 s, while the venous phase images were obtained with a post-injection delay of 60–70 s.

### Image segmentation and radiomics extraction

**Fig. 2:**
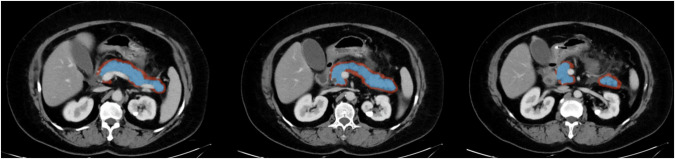
Example of regions of interest (ROIs) for acute pancreatitis. The pancreatic ROIs (blue area) were manually delineated along the edge of the pancreatic parenchyma, and the peripancreatic ROIs (red area) were delineated by expanding the pancreatic ROIs by 5 mm towards the peripancreatic area

Two experienced radiologists (X.L.W. and D.L., with 8 and 10 years of experience in the field of abdominal imaging, respectively) conducted a joint review of all abdominal CT images and reached a consensus after discussing if there was disagreement regarding the CT features. CT features were evaluated, including pancreatic enlargement, peripancreatic inflammation, peripancreatic effusion, peripancreatic gas, pancreatic necrosis, extrapancreatic complications such as pleural effusion, peritoneal effusion, vascular or gastrointestinal complications. All extrapancreatic complications were lumped together and considered as a single variable that was either present or absent. The CT images of the venous phase were imported into the uAI research portal (uRP) (version 211230), and radiomic features were extracted from regions of interest (ROIs) via this software. The pancreatic ROIs were manually delineated by the two radiologists on each axial slice along the edge of the pancreatic parenchyma covering the whole pancreatic region. The corresponding peripancreatic ROIs were delineated by expanding the pancreatic ROI by 5 mm towards the peripancreatic area (the peripancreatic area encompassed a 5 mm distance from the pancreatic surface, excluding the pancreatic parenchyma area, blood vessels, bile ducts, peripancreatic lymph nodes and organs) (Fig. 2). After the image segmentation, to mitigate potential influences stemming from image and radiomics features, all images were resampled using a linear interpolation algorithm to achieve voxel dimensions of 1 mm × 1 mm × 1 mm [20]. The time spent intermittently on the entire process of segmentation and resampling by the researchers amounted to approximately 2 months. Furthermore, the radiomics features were normalized by *Z*-score standardization.

### Intra- and interobserver agreement

A set of 50 patient images was randomly selected to be assessed. Two radiologists (X.L.W. and D.L.) independently delineated pancreatic and peripancreatic ROIs to evaluate the interobserver agreement. To assess intraobserver agreement, a radiologist (X.L.W.) delineated these ROIs again with a gap of two weeks following the same procedure. The features obtained from the two extractions were then compared.

### Radiomics and clinical feature selection and model construction

The selection of optimal radiomics features was performed through a sequential process involving three algorithms. Firstly, the variance threshold method was applied with a threshold set at 0.8, and then the Select K-Best method with ANOVA *F*-value (*p* < 0.05) was used to choose features. Finally, the least absolute shrinkage and selection operator (LASSO) method was utilized with parameters set at k-fold = 5 and alpha = 0.00236. Radscore was calculated by linearly multiplying the optimal radiomics features with their corresponding LASSO coefficients. The logistic regression classifiers were used to construct the models. The variance inflation factor (VIF) was used to assess the presence of multicollinearity among clinical variables and CT features. Variables with VIF > 10, including gender, severity of AP, pancreatic enlargement, and peripancreatic inflammation, were removed. The remaining variables were then included in the univariate and multivariate logistic regression analyses to identify independent risk factors associated with poor prognosis. Based on the independent risk factors and the optimal radiomics features, the clinical, pancreatic, peripancreatic, radiomics, and combined models were constructed. Additionally, the data of the test set was used to independently evaluate the performance of the combined model.

### Statistical analysis

Statistical analyses were performed using SPSS (version 25.0; IBM) and R software (version 3.6.1). Normality testing of continuous variables was performed using the Shapiro–Wilk test. Continuous variables were compared by using the Student’s *t*-test or Mann–Whitney *U*-test. Categorical variables are presented as numbers and percentages. Categorical variables were compared using the chi-square test or Fisher exact test as appropriate. The area under the receiver operating characteristic curve (AUC) was calculated to evaluate the predictive ability. The calibration curve and the decision curve analyses (DCA) were used to evaluate the calibration and clinical practicability of the combined model using the R software. The AUCs of the five models were compared using the DeLong test. The inter-class correlation coefficient (ICC) was calculated by a two-way mixed-effects model to quantify the consistency of feature extraction [21], ICC > 0.75 in both test-retest and inter-reader analyses is considered indicative of good consistency. *p* < 0.05 was considered statistically significant.

## Results

### Patient characteristics and clinical model

All 344 patients in a first attack of AP were enrolled in the present study. In order to provide an unbiased evaluation of a final model fit on the training set, this study adopted a split ratio of 6: 2: 2 to divide all patient datasets into training (*n* = 206), validation (*n* = 69), and test (*n* = 69) sets according to the common experience [22]. Among these patients, there were 171 males and 173 females. The average age of all patients was 51 ± 15 years. Detailed baseline clinical and CT imaging characteristics of patients are summarized in Table 1. The results of univariate and multivariate regression analyses in the training and validation sets indicated that CRP (*p* < 0.01), WBC (*p* = 0.040), peripancreatic effusion (*p* = 0.019), and extrapancreatic complications (*p* = 0.012) were independently associated with the poor prognosis (Table 2). Therefore, the above independent risk factors were incorporated to build the clinical model.

**Table 1:** Clinical and imaging characteristics of patients with acute pancreatitis in the training, validation, and test sets

Characteristics	Training (*n* = 206)	Validation (*n* = 69)	Test (*n* = 69)	^a^*p-*value	^b^*p-*value
Age, median (IQR)	49 (41, 61)	50 (39, 62)	51 (38, 65)	0.548	0.533
Gender, *n* (%)				0.102	0.553
Male	96 (46.6)	40 (58)	35 (50.7)		
Female	110 (53.4)	29 (42)	34 (49.3)		
Diabetes, *n* (%)				0.227	0.667
No	169 (82)	52 (75.4)	55 (79.7)		
Yes	37 (18)	17 (24.6)	14 (20.3)		
Severity of AP, *n* (%)				0.57	0.274
MAP	59 (28.6)	17 (24.6)	14 (20.3)		
MSAP	133 (64.6)	49 (71)	52 (75.4)		
SAP	14 (6.8)	3 (4.3)	3 (4.3)		
Prognosis, *n* (%)				0.91	0.921
Good	124 (60.2)	41 (59.4)	42 (60.9)		
Poor	82 (39.8)	28 (40.6)	27 (39.1)		
BISAP, median (IQR)	1 (0, 2)	1 (0, 1)	1 (0, 2)	0.287	0.821
CRP, median (IQR)	34.1 (7.7, 107.3)	80.6 (10.2, 166.3)	24.0 (7.4, 111.5)	0.039	0.604
PCT, median (IQR)	0.12 (0.05, 0.44)	0.14 (0.06, 0.38)	0.10 (0.05, 0.34)	0.391	0.568
WBC, mean ± SD	12.8 ± 4.2	12.7 ± 3.8	13.4 ± 4.8	0.771	0.643
Etiology, *n* (%)				0.969	0.393
Biliary	46 (22.3)	17 (24.6)	22 (31.9)		
Alcoholic	11 (5.3)	4 (5.8)	2 (2.9)		
Hyperlipidemic	99 (48.1)	31 (44.9)	29 (42)		
Other	50 (24.3)	17 (24.6)	16 (23.2)		
Pancreatic enlargement, *n* (%)				0.171	0.746
No	11 (5.3)	1 (1.4)	3 (4.3)		
Yes	195 (94.7)	68 (98.6)	66 (95.7)		
Pancreatic necrosis, *n* (%)				0.586	0.33
No	190 (92.2)	65 (94.2)	61 (88.4)		
Yes	16 (7.8)	4 (5.8)	8 (11.6)		
Peripancreatic inflammation, *n* (%)				0.415	0.562
No	1 (0.5)	1 (1.4)	0 (0)		
Yes	205 (99.5)	68 (98.6)	69 (100)		
Peripancreatic effusion, *n* (%)				0.908	0.237
No	79 (38.3)	27 (39.1)	21 (30.4)		
Yes	127 (61.7)	42 (60.9)	48 (69.6)		
Peripancreatic gas, *n* (%)				0.562	0.415
No	205 (99.5)	69 (100)	68 (98.6)		
Yes	1 (0.5)	0 (0)	1 (1.4)		
Extrapancreatic complications, *n* (%)				0.895	0.57
No	148 (71.8)	49 (71)	52 (75.4)		
Yes	58 (28.2)	20 (29)	17 (24.6)		

Note: Variables are presented as mean ± SD or median (IQR) for continuous data and *n* (%) for categorical data

The prognosis was based on the follow-up CT or clinical data (the occurrence of infection or persistent organ failure during hospitalization)

*BISAP* bedside index for severity in acute pancreatitis, *CRP* C-reactive protein, *PCT* procalcitonin, *WBC* white blood cell, *MAP* mild acute pancreatitis, *MSAP* moderately severe acute pancreatitis, *SAP* severe acute pancreatitis

^a^ *p-*value for training set vs. validation set

^b^ *p*-value for training set vs. test set

**Table 2:** Univariate and multivariate logistic regression analyses of clinical characteristics in patients with acute pancreatitis

	Univariate			Multivariate		
	*β*	OR (95% CI)	*p-*value	*β*	OR (95% CI)	*p-*value
Age	−0.015	0.985 (0.966, 1.004)	0.132			
BISAP	0.762	2.143 (1.541, 3.073)	**<** **0.01**	0.311	1.365 (0.869, 2.177)	0.182
CRP	0.01	1.01 (1.006, 1.015)	**<** **0.01**	0.013	1.013 (1.007, 1.019)	**<** **0.01**
PCT	0.082	1.085 (0.966, 1.301)	0.257			
WBC	0.134	1.143 (1.063, 1.236)	**<** **0.01**	0.098	1.103 (1.002, 1.218)	**0.048**
Etiology	0.079	1.083 (0.829, 1.421)	0.562			
Diabetes	0.497	1.644 (0.79, 3.426)	0.181			
Peripancreatic effusion	1.917	6.797 (3.414, 14.465)	**<** **0.01**	1.041	2.832 (1.202, 6.949)	**0.019**
Peripancreatic gas	14.98	3,205,140.755 (0.6410821.510)	0.986			
Pancreatic necrosis	2.345	10.431 (2.735, 68.378)	**0.003**	1.133	3.106 (0.656, 22.77)	0.190
Extrapancreatic complications	2.242	9.415 (4.645, 20.196)	**<** **0.01**	1.211	3.357 (1.321, 8.844)	**0.012**

Note: Bold values denote statistical significance at the *p* < 0.05 level

*BISAP* bedside index for severity in acute pancreatitis, *CRP* C-reactive protein, *PCT* procalcitonin, *WBC* white blood cell

### Radiomics features and radiomics-based models

A total of 2264 radiomics features were meticulously extracted, consisting of 104 original image features and 2160 filter features from pancreatic and peripancreatic ROIs. Following the simultaneous exclusion of identical features based on both intra- and interobserver agreement (Electronical Supplementary Material), a final set of 1961 radiomics features from the pancreas and 1794 radiomics features from the peripancreatic ROIs were retained for subsequent dimensionality reduction. After applying a sequential dimensionality reduction, 8 optimal radiomics features were identified from the pancreatic ROIs, while 6 optimal radiomics features were selected from the peripancreatic ROIs (Table 3). The pancreatic and peripancreatic radiomics features were separately utilized to construct the pancreatic and peripancreatic models. Furthermore, all 14 optimal radiomics features were combined to construct a radiomics model and further processed to calculate the radscore (Electronic Supplementary Material).

**Table 3:** The final pancreatic and peripancreatic radiomics features selected in the radscore

Pancreatic radiomics features (*n* = 8)	Peripancreatic radiomics features (*n* = 6)
boxsigmaimage_firstorder_RobustMeanAbsoluteDeviation	log_firstorder_log-sigma-2-0-mm-3D-Median
boxsigmaimage_glrlm_GrayLevelNonUniformityNormalized	wavelet_firstorder_wavelet-LLH-InterquartileRange
log_firstorder_log-sigma-1-0-mm-3D-Mean	wavelet_glrlm_wavelet-LLH-GrayLevelNonUniformityNormalized
log_firstorder_log-sigma-2-0-mm-3D-10Percentile	specklenoise_glcm_Idm
log_firstorder_log-sigma-4-0-mm-3D-RootMeanSquared	specklenoise_glrlm_LongRunEmphasis
log_gldm_log-sigma-4-0-mm-3D-SmallDependenceEmphasis	specklenoise_gldm_LargeDependenceEmphasis
wavelet_glcm_wavelet-LLL-InverseVariance	
wavelet_glrlm_wavelet-LLH-RunEntropy	

**Fig. 3:**
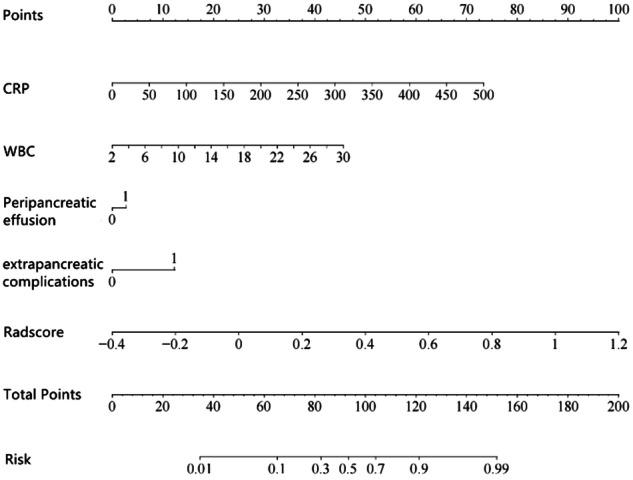
The nomogram incorporated radscore and clinical features for the prediction of prognosis in patients with acute pancreatitis

In addition, in order to incorporate both radiomics and clinical features, the combined model was constructed by integrating the radscore with the aforementioned independent clinical risk factors (CRP, WBC, peripancreatic effusion, and extrapancreatic complications). Figure 3 shows the nomogram incorporated radscore and clinical features for the prediction of AP prognosis.

### Performance evaluation and validation

**Fig. 4:**
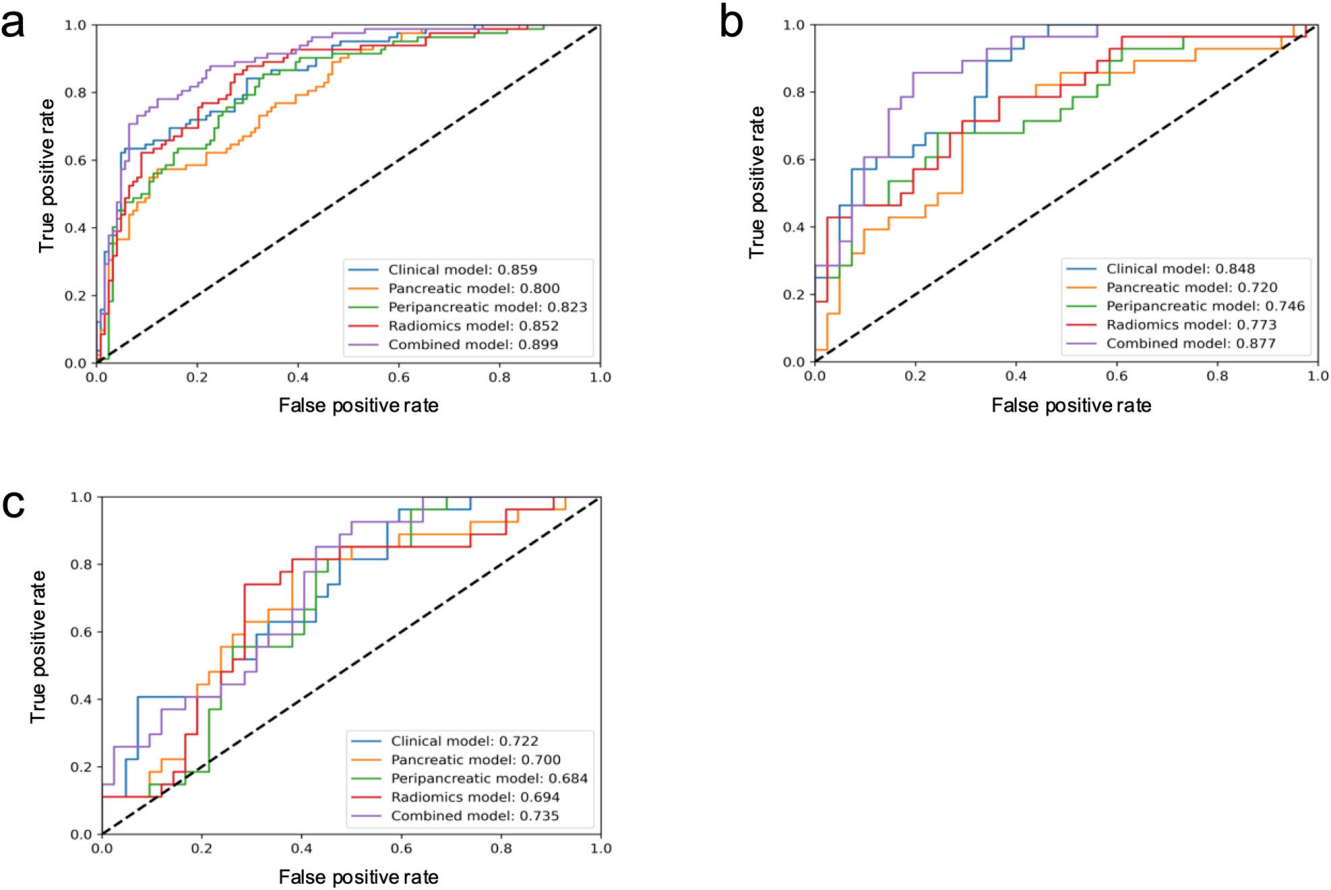
The receiver operating characteristic curves of the five models in the training set (**a**), validation set (**b**), and test set (**c**)

**Fig. 5:**
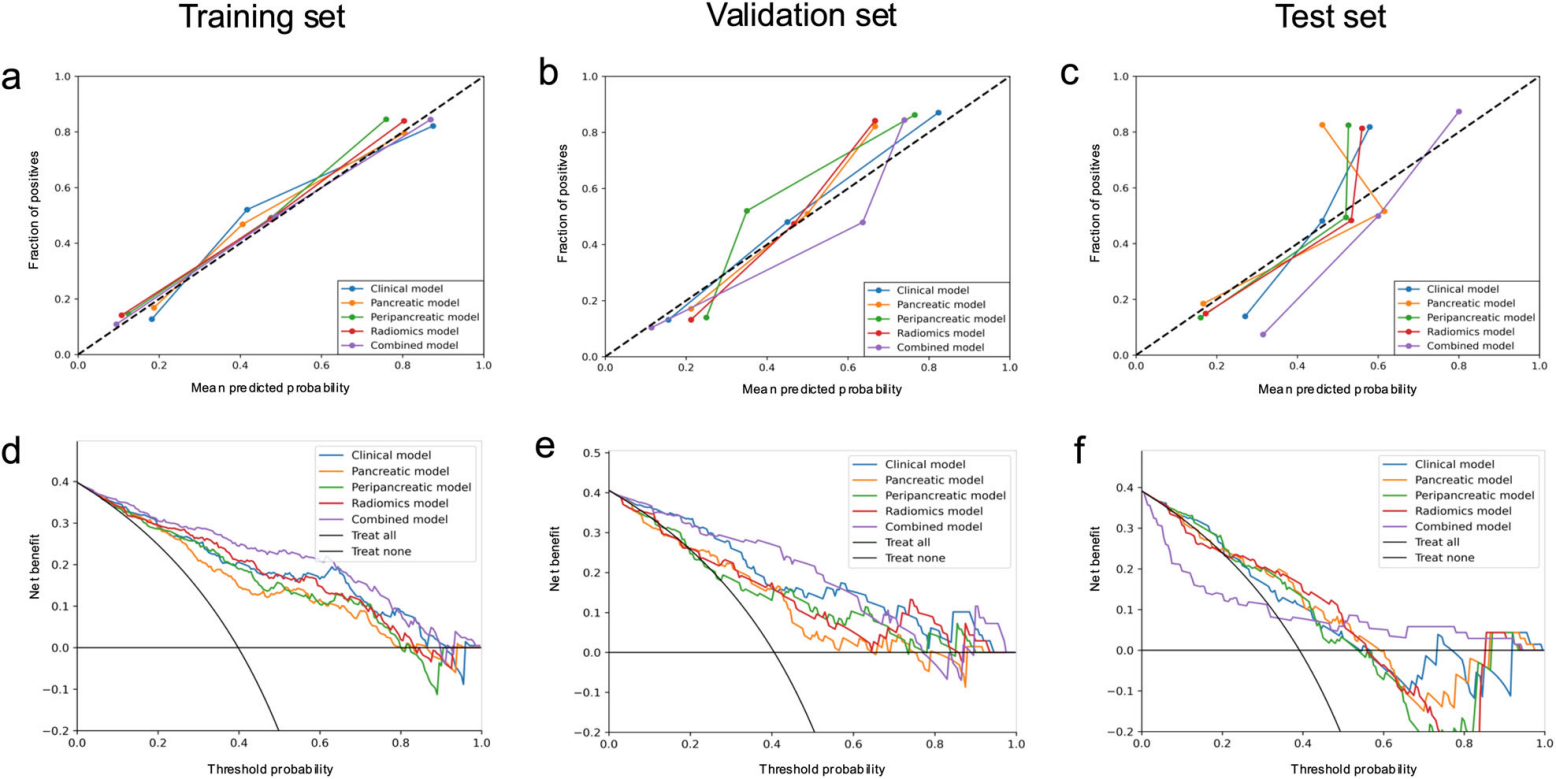
Calibration curves of the clinical, pancreatic, peripancreatic, radiomics, and combined models in the training set (**a**) and validation set (**b**). The dotted line indicates the optimal prediction and the solid line represents the real predictive ability of the model. The closer the solid line approaches the dotted line, the better the predictive efficacy of the model is. The calibration curves demonstrated good calibration of the combined model in both the training and validation sets. Decision curve analysis for the five models in the training set (**d**) and validation set (**e**). Calibration curves (**c**) and decision curve analysis (**f**) for the five models in the test set. The *x*- and *y*-axis indicate threshold probability and the net benefit, respectively. The combined model showed reasonable net benefits in all models

The training set showed AUCs of 0.859 for the clinical model, 0.800 for the pancreatic model, 0.823 for the peripancreatic model, 0.852 for the radiomics model, and 0.899 for the combined model (Table 4 and Fig. 4a). In the validation set, the corresponding AUCs were 0.848, 0.720, 0.746, 0.773, and 0.877, respectively (Table 4 and Fig. 4b). Notably, the AUC of the combined model for predicting the prognosis of AP was higher than those of the pancreatic model, peripancreatic model, and radiomics model in both the training and validation sets (all *p* < 0.05). Comparing the training set, the combined model showed superior predictive performance compared to the clinical model (*p* < 0.05), while no significant difference was observed in the validation set (*p* > 0.05). Further, the combined model indicated its reasonable predictive performance with the AUC value of 0.735 (0.619–0.852) in the test set (Table 4 and Fig. 4c). The calibration curves demonstrated good calibration of the combined model in both the training and validation sets (Fig. 5a, b). In addition, the DCA indicated that the combined model displayed the greatest net benefit in all models across the relevant threshold range in both the training and validation sets (Fig. 5d, e). The calibration and DCA curves in the test set are shown in Fig. 5c, f, respectively.

**Table 4:** Performance of the five models in the training, validation, and test sets

	AUC (95% CI)	Sensitivity (%)	Specificity (%)	Accuracy (%)	Precision (%)
Training set					
Clinical model	0.859 (0.808, 0.909)	69.5	83.1	77.7	73.1
Pancreatic model	0.800 (0.740, 0.860)	57.3	83.9	73.3	70.1
Peripancreatic model	0.823 (0.765, 0.881)	63.4	83.9	75.7	72.2
Radiomics model	0.852 (0.799, 0.905)	69.5	83.1	77.7	73.1
Combined model	0.899 (0.856, 0.941)	78.0	87.1	83.5	80.0
Validation set					
Clinical model	0.848 (0.760, 0.936)	60.7	82.9	73.9	70.8
Pancreatic model	0.720 (0.594, 0.846)	50.0	73.2	63.8	56.0
Peripancreatic model	0.746 (0.625, 0.867)	60.7	75.6	69.6	63.0
Radiomics model	0.773 (0.658, 0.887)	57.1	75.6	68.1	61.5
Combined model	0.877 (0.797, 0.957)	75.0	85.4	81.2	77.8
Test set					
Clinical model	0.722 (0.602, 0.842)	48.1	73.8	63.8	54.2
Pancreatic model	0.700 (0.573, 0.827)	56.6	73.8	66.7	57.7
Peripancreatic model	0.684 (0.559, 0.809)	55.6	69.0	63.8	53.6
Radiomics model	0.694 (0.564, 0.824)	63.0	71.4	68.1	60.7
Combined model	0.735 (0.619, 0.852)	25.9	92.9	66.7	70.0

*AUC* area under the receiver operating characteristic curve

## Discussion

In the current study, clinical and 4 radiomics-derived models (pancreatic, peripancreatic, radiomics, and combined models) to predict AP prognosis were developed and evaluated. Among these radionics-derived models, the combined model integrated the radscore with clinically independent risk factors. The main findings of this study are as follows: (1) the combined model was superior in predicting the prognosis of AP than the pancreatic, peripancreatic, and radiomics models in both the training set and the validation set; (2) in the training set, the combined model exhibited better predictive performance compared to the clinical model, but no significant difference in the validation set; (3) the combined model exhibited a good calibration and significant clinical applicability. Our findings suggested that integrating radiomics and clinical features has the potential to improve performance for predicting the prognosis of AP.

When the body undergoes an inflammatory reaction, the levels of WBC and CRP in the peripheral blood increase. Previous studies have shown that patients with severe AP tend to have elevated WBC and CRP levels, and both are closely associated with patient mortality [23–25]. Moreover, literature has demonstrated a strong correlation between pleural effusion and the severity and prognosis of AP; patients with peritoneal effusion often experience a higher incidence of organ failure and pancreatic necrosis [26–28]. Similarly, this study revealed that extrapancreatic complications of AP patients mainly manifested as pleural effusion and peritoneal effusion. The incidence of extrapancreatic complications in the group with a poor prognosis was significantly higher compared to the group with a good prognosis (74.4% vs. 25.6%), which might be explained by the activation of pancreatic enzymes within the pancreatic vesicles and the potential destruction of pancreatic ducts during AP. Initially, inflammatory pancreatic fluid leaks into the peripancreatic area, causing acute fluid accumulation, it subsequently diffuses through the retroperitoneal space and penetrates the peritoneum, resulting in peritoneal effusion. The accumulation of peritoneal effusion can lead to intra-abdominal hypertension, which in turn affects renal function [29, 30]. Furthermore, the inflammatory pancreatic fluid may lead to pancreatic pleural fistula or enter the thoracic cavity through the lymphatic plexus of the diaphragm, resulting in pleural effusion and a subsequent reduction in lung function [29, 30]. Thus the predictive performance of the clinical model is likely attributed to the aforementioned association between the clinical features and AP prognosis. However, the clinical model exhibited low specificity and sensitivity due to missing important imaging information. Other scoring systems, including the Acute Physiology and Chronic Health Evaluation (APACHE) score [31], BISAP score [32], and MCTSI [33], are commonly used to evaluate the condition of AP patients in clinical practice, but MCTSI based on visual morphological changes may be susceptible to subjective interpretation by radiologists, and the accuracy and sensitivity of these scoring systems are not particularly high.

Radiomics analysis allows the extraction of texture feature parameters, thereby uncovering microscopic information ignored by the naked eye, which offers more favorable decision support in clinical practice [34]. Previous studies have reported that extracting radiomics features from CT or MRI can provide additional information for differentiating phenotypes of pancreatitis or predicting AP prognosis [35–39]. Zhou et al constructed MRI-based radiomics models to predict early extrapancreatic necrosis and demonstrated the MRI-based radiomics models had better predictive performance compared to the clinical model [35]. Lin et al established an MRI-based radiomics model to predict the severity of AP and validated its excellent performance than other scoring systems [36]. A recent study investigated the predictive value of an MRI-based radiomics model in forecasting the risk of recurrence [38]. Mashayekhi et al demonstrated the ability of CT-based radiomics features in differentiating functional abdominal pain, recurrent AP, and chronic pancreatitis [39]. Although previous studies have confirmed the superiority of radiomics features in predicting the prognosis or severity of AP, these models did not incorporate clinical information. In the current study, we harnessed the advantages of radiomics features and integrated them with clinical features to optimize predictive performance. Our findings also demonstrated the superiority of the combined model using a visual nomogram tool in predicting AP prognosis, surpassing both the clinical model and the three other models that rely solely on CT radiomics.

It is worth noting that during the attack of AP, the pancreas undergoes morphological changes as a result of self-digestion. In addition, inflammatory pancreatic fluid can readily spread to the peripancreatic area in the early stages, potentially affecting the peripancreatic fatty space. Therefore, in this current study, radiomics features were extracted from CT imaging of both the pancreatic parenchyma and peripancreatic regions. These pancreatic and peripancreatic features were further combined to calculate the radscore, which can provide a more comprehensive assessment of pancreatic and peripancreatic inflammation.

Our study has several limitations. Firstly, it is important to note that this study is a retrospective study, which may potentially introduce selection bias in case selection. Secondly, the combined model developed in this study was based on data from a single center, thereby its reliability and reproducibility need to be further verified through multi-center studies with larger sample sizes. Further, the conclusions of the current study might not generalize patients who present with AP but were excluded from this study, especially in terms of mild AP patients, missing clinical data, and/or the failure to repeat a CT 7–10 days after the initial CT. This lack of patient diversity in the training set or selection bias can limit the generalizability of radiomics findings. To mitigate this bias, future radiomics studies could involve integrating data from patients who undergo ultrasound or are diagnosed based on blood tests, potentially by linking imaging data. Variability across imaging protocols can also make it difficult to generalize findings. Limitations also include the need for manual segmentation, especially for practices without abdominal imaging subspecialists. The evaluation of models’ performance might be determined by sample size, different set pairs, and the complexity or difficulty of the learning task. Consequently, it is essential to conduct external validation to assess the performance of these models in the later study.

## Conclusions

CT radiomics-based combined model integrated with clinical features, showed superior predictive performance in evaluating the prognosis of AP. It might supplement additional imaging information missed by the clinical model to some extent, thereby providing a more comprehensive assessment of AP prognosis. This enhanced assessment facilitates the optimization of clinical decision-making for individuals at risk of developing poor prognoses, allowing for more tailored therapeutic strategies in clinical routine.

## Supplementary information

ELECTRONIC SUPPLEMENTARY MATERIAL

## Data Availability

The datasets used and/or analyzed during the current study are available from the corresponding author upon reasonable request.

## Abbreviations

AP: Acute pancreatitis
AUC: Area under the receiver operating characteristic curve
BISAP: Bedside Index for Severity in Acute Pancreatitis
DCA: Decision curve analysis
ICC: Inter-class correlation coefficient
LASSO: Least absolute shrinkage and selection operator
MAP: Mild acute pancreatitis
MCTSI: Modified computed tomography severity index
MSAP: Moderately severe acute pancreatitis
ROI: Region of interest
SAP: Severe acute pancreatitis
VIF: Variance inflation factor

## References

[1] Boxhoorn L, Voermans RP, Bouwense SA et al (2020) Acute pancreatitis. Lancet 396:726–734 32891214 10.1016/S0140-6736(20)31310-6

[2] Petrov MS, Yadav D (2019) Global epidemiology and holistic prevention of pancreatitis. Nat Rev Gastroenterol Hepatol 16:175–184 30482911 10.1038/s41575-018-0087-5 PMC6597260

[3] van Dijk SM, Hallensleben NDL, van Santvoort HC et al (2017) Acute pancreatitis: recent advances through randomised trials. Gut 66:2024–2032 28838972 10.1136/gutjnl-2016-313595

[4] Schepers NJ, Bakker OJ, Besselink MG et al (2019) Impact of characteristics of organ failure and infected necrosis on mortality in necrotising pancreatitis. Gut 68:1044–1051 29950344 10.1136/gutjnl-2017-314657

[5] Fta B, Hlb C, Liang WB et al (2020) The diagnostic value of serum C-reactive protein, procalcitonin, interleukin-6 and lactate dehydrogenase in patients with severe acute pancreatitis—ScienceDirect. Clin Chim Acta 510:665–670 32828732 10.1016/j.cca.2020.08.029

[6] Balthazar EJ (2002) Acute pancreatitis: assessment of severity with clinical and CT evaluation. Radiology 223:603–613 12034923 10.1148/radiol.2233010680

[7] Rocha APC, Schawkat K, Mortele KJ (2020) Imaging guidelines for acute pancreatitis: when and when not to image. Abdom Radiol (NY) 45:1338–1349 31712865 10.1007/s00261-019-02319-2

[8] Porter KK, Cason DE, Morgan DE (2018) Acute pancreatitis: how can MR imaging help. Magn Reson Imaging Clin N Am 26:439–450 30376980 10.1016/j.mric.2018.03.011

[9] Barral M, Taouli B, Guiu B et al (2015) Diffusion-weighted MR imaging of the pancreas: current status and recommendations. Radiology 274:45–63 25531479 10.1148/radiol.14130778

[10] Iranmahboob AK, Kierans AS, Huang C, Ream JM, Rosenkrantz AB (2017) Preliminary investigation of whole-pancreas 3D histogram ADC metrics for predicting progression of acute pancreatitis. Clin Imaging 42:172–177 28068586 10.1016/j.clinimag.2016.12.007

[11] Chen Y, Chen TW, Wu CQ et al (2019) Radiomics model of contrast-enhanced computed tomography for predicting the recurrence of acute pancreatitis. Eur Radiol 29:4408–4417 30413966 10.1007/s00330-018-5824-1

[12] Shi N, Zhang X, Zhu Y et al (2022) Predicting persistent organ failure on admission in patients with acute pancreatitis: development and validation of a mobile nomogram. HPB (Oxford) 24:1907–1920 35750613 10.1016/j.hpb.2022.05.1347

[13] Zhong J, Hu Y, Xing Y et al (2022) A systematic review of radiomics in pancreatitis: applying the evidence level rating tool for promoting clinical transferability. Insights Imaging 13:139 35986798 10.1186/s13244-022-01279-4 PMC9391628

[14] Banks PA, Bollen TL, Dervenis C et al (2013) Classification of acute pancreatitis-2012: revision of the Atlanta classification and definitions by international consensus. Gut 62:102–111 23100216 10.1136/gutjnl-2012-302779

[15] Gomatos IP, Xiaodong X, Ghaneh P et al (2014) Prognostic markers in acute pancreatitis. Expert Rev Mol Diagn 14:333–346 24649820 10.1586/14737159.2014.897608

[16] Abu Omar Y, Attar BM, Agrawal R et al (2019) Revised Marshall score: a new approach to stratifying the severity of acute pancreatitis. Dig Dis Sci 64:3610–3615 31286346 10.1007/s10620-019-05719-y

[17] Petrov MS, Shanbhag S, Chakraborty M, Phillips AR, Windsor JA (2010) Organ failure and infection of pancreatic necrosis as determinants of mortality in patients with acute pancreatitis. Gastroenterology 139:813–820 20540942 10.1053/j.gastro.2010.06.010

[18] Marshall JC, Cook DJ, Christou NV, Bernard GR, Sprung CL, Sibbald WJ (1995) Multiple organ dysfunction score: a reliable descriptor of a complex clinical outcome. Crit Care Med 23:1638–1652 7587228 10.1097/00003246-199510000-00007

[19] Choi YR, Chung JW, Yu MH, Lee M, Kim JH (2018) Diagnostic accuracy of contrast-enhanced dynamic CT for small hypervascular hepatocellular carcinoma and assessment of dynamic enhancement patterns: results of two-year follow-up using cone-beam CT hepatic arteriography. PLoS One 13:e0203940 30231076 10.1371/journal.pone.0203940 PMC6145528

[20] Seppa M (2007) High-quality two-stage resampling for 3-D volumes in medical imaging. Med Image Anal 11:346–360 17482501 10.1016/j.media.2007.03.002

[21] Koo TK, Li MY (2016) A guideline of selecting and reporting intraclass correlation coefficients for reliability research. J Chiropr Med 15:155–163 27330520 10.1016/j.jcm.2016.02.012 PMC4913118

[22] Kim TM, Choi SJ, Ko JY et al (2023) Fully automatic volume measurement of the adrenal gland on CT using deep learning to classify adrenal hyperplasia. Eur Radiol 33:4292–4302 36571602 10.1007/s00330-022-09347-5

[23] Liu CN, Chen S, Chen H et al (2019) Peak urea level, leukocyte count and use of invasive ventilation as risk factors of mortality in acute pancreatitis: a retrospective study. PLoS One 14:e0216562 31075129 10.1371/journal.pone.0216562 PMC6510417

[24] Karakulak S, Narcı H, Ayrık C, Erdoğan S, Üçbilek E (2021) The prognostic value of immature granulocyte in patients with acute pancreatitis. Am J Emerg Med 44:203–207 32220526 10.1016/j.ajem.2020.03.028

[25] Dancu GM, Popescu A, Sirli R et al (2021) The BISAP score, NLR, CRP, or BUN: which marker best predicts the outcome of acute pancreatitis? Medicine (Baltimore) 100:e28121 34941057 10.1097/MD.0000000000028121 PMC8702250

[26] Yan G, Li H, Bhetuwal A et al (2021) Pleural effusion volume in patients with acute pancreatitis: a retrospective study from three acute pancreatitis centers. Ann Med 53:2003–2018 34727802 10.1080/07853890.2021.1998594 PMC8567956

[27] Liu ZY, Tian L, Sun XY et al (2022) Development and validation of a risk prediction score for the severity of acute hypertriglyceridemic pancreatitis in Chinese patients. World J Gastroenterol 28:4846–4860 36156930 10.3748/wjg.v28.i33.4846 PMC9476862

[28] Samanta J, Rana A, Dhaka N et al (2019) Ascites in acute pancreatitis: not a silent bystander. Pancreatology 19:646–652 31301995 10.1016/j.pan.2019.06.004

[29] Kumar P, Gupta P, Rana S (2018) Thoracic complications of pancreatitis. JGH Open 3:71–79 10.1002/jgh3.12099 PMC6386740 30834344

[30] Zeng QX, Wu ZH, Huang DL, Huang YS, Zhong HJ (2021) Association between ascites and clinical findings in patients with acute pancreatitis: a retrospective study. Med Sci Monit 27:e933196 34737257 10.12659/MSM.933196 PMC8577037

[31] Mok SR, Mohan S, Elfant AB, Judge TA (2015) The acute physiology and chronic health evaluation IV, a new scoring system for predicting mortality and complications of severe acute pancreatitis. Pancreas 44:1314–1319 26418901 10.1097/MPA.0000000000000432

[32] Hagjer S, Kumar N (2018) Evaluation of the BISAP scoring system in prognostication of acute pancreatitis—a prospective observational study. Int J Surg 54:76–81 29684670 10.1016/j.ijsu.2018.04.026

[33] Mortele KJ, Wiesner W, Intriere L et al (2004) A modified CT severity index for evaluating acute pancreatitis: improved correlation with patient outcome. AJR Am J Roentgenol 183:1261–1265 15505289 10.2214/ajr.183.5.1831261

[34] Lambin P, Leijenaar RTH, Deist TM et al (2017) Radiomics: the bridge between medical imaging and personalized medicine. Nat Rev Clin Oncol 14:749–762 28975929 10.1038/nrclinonc.2017.141

[35] Zhou T, Xie CL, Chen Y et al (2021) Magnetic resonance imaging-based radiomics models to predict early extrapancreatic necrosis in acute pancreatitis. Pancreas 50:1368–1375 35041335 10.1097/MPA.0000000000001935

[36] Lin Q, Ji YF, Chen Y et al (2020) Radiomics model of contrast-enhanced MRI for early prediction of acute pancreatitis severity. J Magn Reson Imaging 51:397–406 31132207 10.1002/jmri.26798

[37] Hu Y, Liu N, Tang L et al (2022) Three-dimensional radiomics features of magnetic resonance T2-weighted imaging combined with clinical characteristics to predict the recurrence of acute pancreatitis. Front Med 9:777368 10.3389/fmed.2022.777368 PMC8960240 35360712

[38] Tang L, Ma L, Chen Y et al (2023) Radiomics analysis of contrast-enhanced T1W MRI: predicting the recurrence of acute pancreatitis. Sci Rep 13:2762 36797285 10.1038/s41598-022-13650-y PMC9935887

[39] Mashayekhi R, Parekh VS, Faghih M, Singh VK, Jacobs MA, Zaheer A (2020) Radiomic features of the pancreas on CT imaging accurately differentiate functional abdominal pain, recurrent acute pancreatitis, and chronic pancreatitis. Eur J Radiol 123:108778 31846864 10.1016/j.ejrad.2019.108778 PMC7968044

