# Supplementary material for: Integrating CT-based radiomics and clinical features to better predict the prognosis of acute pancreatitis
Source: Insights Imaging. 2025 Jan 9;16:8. doi: 10.1186/s13244-024-01887-2 (PMC11717748; doi:10.1186/s13244-024-01887-2)
Supplement: Supplementary file 1 — ELECTRONIC SUPPLEMENTARY MATERIAL [file 13244_2024_1887_MOESM1_ESM.pdf]

Integrating CT-based Radiomics and Clinical Features to Better Predict the Prognosis of Acute Pancreatitis

ELECTRONIC SUPPLEMENTARY MATERIAL

Intra- and interobserver agreement

For intraobserver agreement, 2003 pancreatic features demonstrated satisfactory consistency (mean ICC: 0.957, range: 0.750-1.000), resulting in the exclusion of 261 features; 2025 peripancreatic features were found satisfactory consistency (mean ICC: 0.927, range: 0.750-1.000) with the exclusion of 239 features. In terms of interobserver agreement, 2084 pancreatic features demonstrated satisfactory consistency (mean ICC: 0.956, range: 0.751-1.000), and 180 features were eliminated; 1794 peripancreatic features showed satisfactory consistency (mean ICC: 0.917, range: 0.750-1.000), and 470 features were excluded.

The formula for calculating the radscore in the combined model

*Radscore = 0.16737707 * log_firstorder_log-sigma-1-0-mm-3D-Mean +*

*0.127280548 * wavelet_glrlm_wavelet-LLH-GrayLevelNonUniformityNormalized +*

*0.09352608 * wavelet_glcm_wavelet-LLL-InverseVariance +*

*0.054193303 * wavelet_glrlm_wavelet-LLH-RunEntropy +*

*0.0502191 * specklenoise_gldm_LargeDependenceEmphasis +*

*0.0183538068 * specklenoise_glcm_Idm +*

*0.0137501648 * boxsigmaimage_firstorder_RobustMeanAbsoluteDeviation +*

*0.0130556943 * specklenoise_glrlm_LongRunEmphasis +*

*-0.02450364 * log_gldm_log-sigma-4-0-mm-3D-SmallDependenceEmphasis +*

*-0.03578913 * wavelet_firstorder_wavelet-LLH-InterquartileRange +*

*-0.0544432253 * boxsigmaimage_glrlm_GrayLevelNonUniformityNormalized +*

*-0.07849399 * log_firstorder_log-sigma-4-0-mm-3D-RootMeanSquared +*

*-0.09357549 * log_firstorder_log-sigma-2-0-mm-3D-Median +*

*-0.270904452 * log_firstorder_log-sigma-2-0-mm-3D-10Percentile +*

0.4
